# Genetic mapping of quantitative trait loci associated with drought tolerance in chickpea (*Cicer arietinum* L.)

**DOI:** 10.1038/s41598-023-44990-y

**Published:** 2023-10-17

**Authors:** Yashwant K. Yadava, Pooja Chaudhary, Sheel Yadav, Aqeel Hasan Rizvi, Tapan Kumar, Rachna Srivastava, K. R. Soren, C. Bharadwaj, R. Srinivasan, N. K. Singh, P. K. Jain

**Affiliations:** 1 ICAR-National Institute for Plant Biotechnology, IARI Campus, New Delhi, 110012 India; 2 Division of Genetics, ICAR-Indian Agricultural Research Institute, New Delhi, 110012 India; 3 ICAR-Indian Institute of Pulses Research, Kanpur, 208024 India

**Keywords:** Agricultural genetics, Genetic linkage study, Genetic markers, Genomics, Genotype, Plant breeding, Plant biotechnology, Plant breeding, Plant genetics, Plant stress responses

## Abstract

Elucidation of the genetic basis of drought tolerance is vital for genomics-assisted breeding of drought tolerant crop varieties. Here, we used genotyping-by-sequencing (GBS) to identify single nucleotide polymorphisms (SNPs) in recombinant inbred lines (RILs) derived from a cross between a drought tolerant chickpea variety, Pusa 362 and a drought sensitive variety, SBD 377. The GBS identified a total of 35,502 SNPs and subsequent filtering of these resulted in 3237 high-quality SNPs included in the eight linkage groups. Fifty-one percent of these SNPs were located in the genic regions distributed throughout the genome. The high density linkage map has total map length of 1069 cm with an average marker interval of 0.33 cm. The linkage map was used to identify 9 robust and consistent QTLs for four drought related traits viz. membrane stability index, relative water content, seed weight and yield under drought, with percent variance explained within the range of 6.29%–90.68% and LOD scores of 2.64 to 6.38, which were located on five of the eight linkage groups. A genomic region on LG 7 harbors quantitative trait loci (QTLs) explaining > 90% phenotypic variance for membrane stability index, and > 10% PVE for yield. This study also provides the first report of major QTLs for physiological traits such as membrane stability index and relative water content for drought stress in chickpea. A total of 369 putative candidate genes were identified in the 6.6 Mb genomic region spanning these QTLs. *In-silico* expression profiling based on the available transcriptome data revealed that 326 of these genes were differentially expressed under drought stress. KEGG analysis resulted in reduction of candidate genes from 369 to 99, revealing enrichment in various signaling pathways. Haplotype analysis confirmed 5 QTLs among the initially identified 9 QTLs. Two QTLs, *qRWC1.1* and *qYLD7.1*, were chosen based on high SNP density. Candidate gene-based analysis revealed distinct haplotypes in *qYLD7.1* associated with significant phenotypic differences, potentially linked to pathways for secondary metabolite biosynthesis. These identified candidate genes bolster defenses through flavonoids and phenylalanine-derived compounds, aiding UV protection, pathogen resistance, and plant structure.The study provides novel genomic regions and candidate genes which can be utilized in genomics-assisted breeding of superior drought tolerant chickpea cultivars.

## Introduction

Chickpea or Bengal gram (*Cicer arietinum* L.) is one of the most important food legume crops cultivated globally^1^. According to FAOSTAT (2020)^2^, among the major legume crops, soybeans lead with a production of approximately 355.37 million tons (mt), followed by peas at 34.99 mt, lima and common beans at 27.41 mt, chickpeas at 15.06 mt, broad beans at 7.47 mt s, cowpeas at 9.04 mt, and pigeonpeas at 5.05 mt. This positions chickpea as the fourth-largest legume crop globally^2^. India is the largest producer (11 million tons) where it produces approximately 73% of the total global production of chickpea^2^. The chickpea production globally is adversely affected by drought stress, which can cause up to 50% yield losses^1^. Prevalence of drought at pod filling stage, commonly referred to as terminal drought, results in reduced flower and pod production and reduced seed size in chickpea plants. With the ongoing global warming, the world is predicted to face even hotter climate and erratic rainfalls in the near future^3,4^. This will lead to an increase in both the incidence and intensity of drought stress during pod filling stage. Hence, it becomes imperative to understand the genetic basis and mechanisms for drought tolerance in chickpea for varietal improvement.

A small genome size of 740 Mbp and the availability of whole genome sequence of chickpea, renders the crop amenable to genomics-assisted breeding for accelerated development of varieties with enhanced drought tolerance. Drought tolerance is a complex phenomenon, which is governed by several genes and involves an intricate network of stress response pathways.

Various physiological and morphological traits have been used to screen drought tolerance in plants, for example water use efficiency (WUE), stomatal conductance (SC), relative water content (RWC), membrane stability index (MSI), canopy temperature depression (CTD), root biomass and architecture, leaf chlorophyll content, leaf rolling, yield and yield components under stress^5,6^. However, the genetics of many of these traits is not sufficiently studied in chickpea. The significance of these traits and their utility in chickpea improvement for drought tolerance has been substantiated with the observation that as many as 13 Meta-QTLs for plant vigor are co-mapped to a previously identified “QTL hotspot” region for drought tolerance^7^. It is believed that a component trait-based selection of plants is more useful inbreeding for improved drought tolerance. Different types of molecular markers have been used to identify the QTL/genes for these traits. A total of 93 QTLs for drought related traits were mapped using SSR markers in a bi-parental mapping population of the cross ILC 588/ ILC 3279^8,9^**.** The “QTL-hotspot” harboring 12 QTLs, governing different drought tolerance traits, with up to 58.2% of the phenotypic variation explained, was mapped on chickpea chromosome 4, by genotyping using 241 SSR markers of an intra-specific RIL population derived from cross ICC 4958 × ICC 1882^10^. This QTL-hotspot became a focal point of investigation for many chickpea geneticists and breeders. The “QTL hotspot” on CaLG04 was narrowed down to about 14 cm, by using additional SNP markers^11^. With the advent of high throughput next generation sequencing (NGS) technologies, single nucleotide polymorphism (SNP) markers have become a favorite choice for genotyping. The advantages of SNP markers include high abundance in the genome, biallelic nature, high reproducibility, low mutation rates and amenability to automation^12^. Some SNPs in the protein coding regions may directly affect the agronomic traits by changing the amino acid sequence of the protein, hence these are known as functional SNPs or perfect markers. Due to these reasons, SNPs are being increasingly used for genotyping in many crops including rice, wheat, barley, sorghum and soybean^13–16^.

Genotyping by Sequencing (GBS) allows bar-coding for large-scale multiplexing of samples to achieve high throughput SNP genotyping with lower cost per sample as compared to high-density SNP chip arrays^13,17,18^. The problem of poor uniformity in genome coverage in GBS analysis has been addressed by restriction site associated DNA (RAD) sequencing, which involves sequential steps of restriction enzyme digestion of genomic DNA for reduced representation, adapter ligation, PCR amplification and sequencing. The approach has been widely used for diversity assessment, trait mapping and genome wide association studies (GWAS) in several crops^19,20^. Further, QTLs for seed traits in chickpea have been mapped using GBS to generate a high-density linkage map of 3,363 SNP markers^12^**.**

Realizing the importance of drought responsive morpho-physiological traits, which allow chickpea plants to withstand drought stress, the present study was undertaken to map the QTLs for drought stress responsive traits viz. membrane stability index (MSI), relative water content (RWC), 100 seed weight (SW) and seed yield per plant (YLD) under drought and to identify candidate genes underlying these QTL regions.

## Results

### SNPs between the parents and annotation of genes

**Figure 1:**
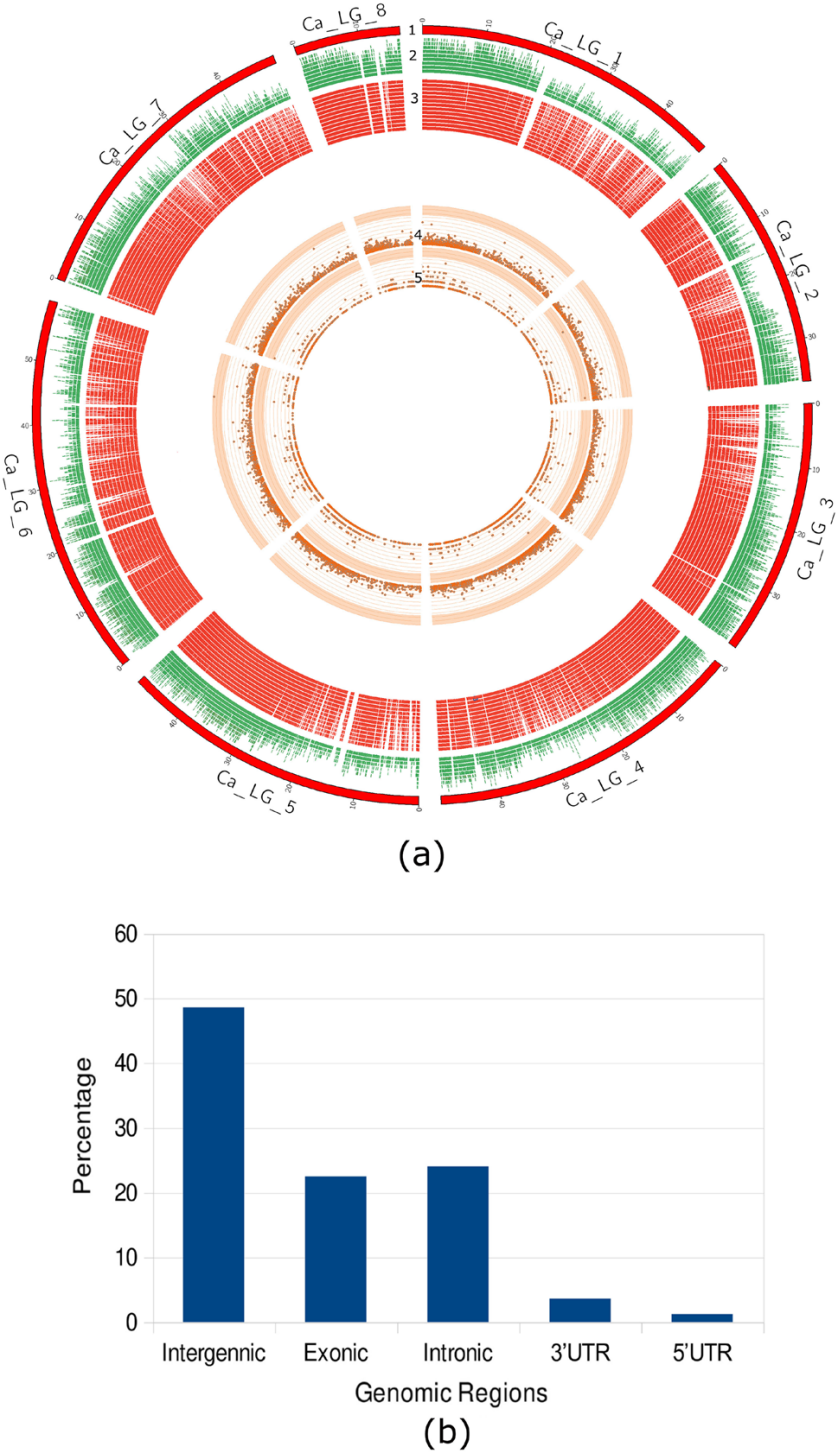
Distribution of SNPs between Pusa 362 and SBD 377 across the chickpea genome. (**a**) Circos diagram depicting chromosome wise distribution of SNPs in 10 kb window size. Track 1 (the outermost circle) represents the 8 chickpea chromosomes (CaLG1-8) in red colour. Tracks 2 , 3, 4, and 5 represent genes, exons, raw SNPs and filtered SNPs, respectively. (**b**) Percent distribution of SNPs in different genomic regions.

A total of 42 Gb data with 209 million reads were generated for the two parents and 186 RILs of the Pusa 362/SBD 377 mapping population. The sequence data has been deposited in the NCBI short reads archive (SRA) database (SRA, 2021) under accession numbers SRR13002229 and SRR13002230. The sequence reads were subjected to quality check (QC). Filtering criterion included removal of barcodes, *Ape*KI restriction enzyme overhangs, and base quality Phred score of ≥ 15 for at least 80% of the bases in a read. After QC, a total of 177 million high quality reads (85% of the original reads) were processed for further analysis. The average number of reads per genotype was 1,125,483 with a variation of 29.88% between individuals. After alignment to the reference genome a total of 35,502 raw SNPs were identified. Of these, 3237 were high quality SNPs identified after stringent filtering, and the sequence information of these 3237 SNPs is provided in Supplementary Table S1. The distribution of SNPs across eight chickpea chromosomes is shown in Table 1, Fig. 1a and Supplementary Table S2. The highest number of SNPs were located on chromosome 6 (636) and the lowest number on chromosome 8 (128). Base transitions were 1.5 times more frequent than transversions.

**Table 1:** Summary statistics of chickpea SNP linkage map constructed using GBS data from ‘Pusa 362/SBD 377’ RIL population.

Chromosome	No. of mapped SNP markers	Map length (cm)	Average map interval (cm)
1	394	144.8	0.367
2	296	109.7	0.370
3	345	119.7	0.347
4	633	147.2	0.232
5	526	144.2	0.274
6	636	178.1	0.280
7	279	144.4	0.517
8	128	81.0	0.632
Total	3237	1069.1	0.330

Genomic distribution of the identified SNPs in intergenic, exonic, intronic and genic UTR regions was determined using the chickpea genome annotation project database (Chickpea Genome Analysis Project, 2013). The highest proportion of the identified SNPs were located in the intergenic regions (48.52%), followed by introns (23.98%), exons (22.48%) and UTRs (5%) (Fig. 1b). There were 784 gene models identified which contained the exonic SNPs (Supplementary Table S3). The pentatricopeptide repeat-containing proteins were found to have the highest number of exonic SNPs followed by genes encoding serine/threonine-protein kinases, E3 ubiquitin protein ligases, ethylene-overproduction proteins and ABC transporter families. Out of the 784 genes, 462 showed a significant matches with entries in the nucleotide data base of NCBI. These genes were classified in to three major GO groups i.e., biological process, molecular function and cellular components (Fig. S1). The genes categorized under the “biological processes” are mostly associated with different metabolic processes. Most of the genes belonging to the “molecular function” group possess ‘catalytic activity’ followed by ‘binding activity’. To identify SNPs in the genes for transcription factors (TF), HMM (Hidden Markov Model) was applied using HMMBUILD (HMMER 3.0, 2015). The most represented families of TFs identified were bHLH (97 genes), MYB (75 genes), NAC (69 genes), ERF (54 genes), WRKY (51 genes), and bZIP (38 genes) (Fig. S2).

### Trait segregation and location of QTLs for drought tolerance

**Figure 2:**
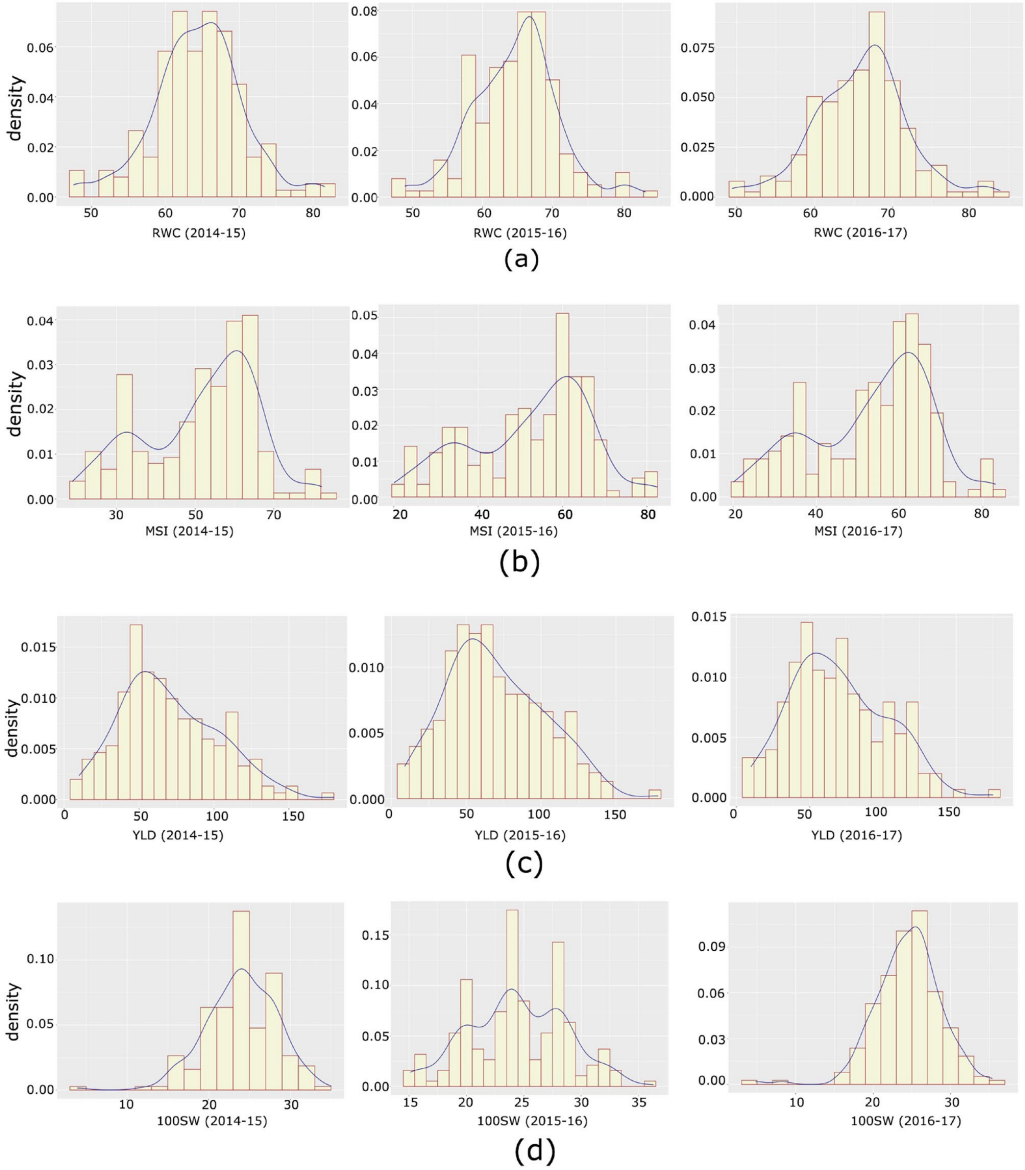
Histogram and phenotypic distribution of (**a**) Relative Water Content (RWC), (**b**) Membrane Stability Index (MSI), (**c**) Yield per plant (YLD), and (**d**) 100 Seed Weight (100SW) in the F11 population derived from the Pusa 362 X SBD377.

**Figure 3:**
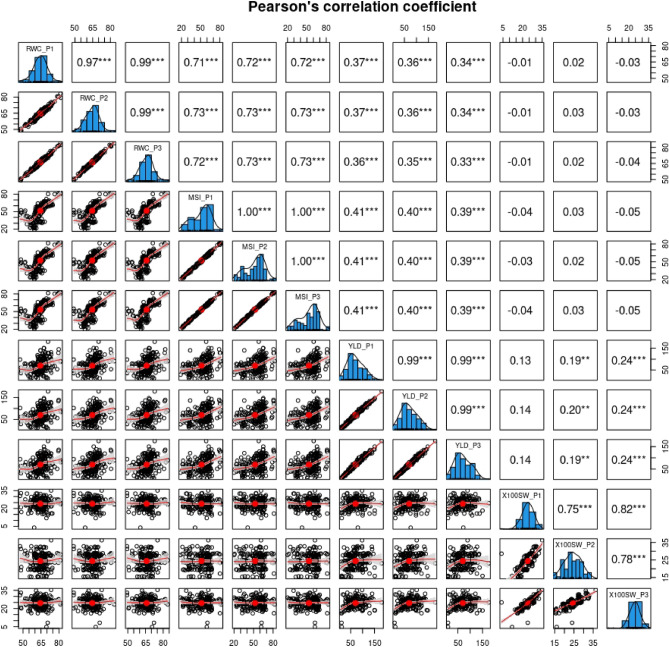
Pearson’s correlation coefficient of relative water content (RWC), membrane stability index (MSI), yield per plant (YLD), and 100 seed weight (100SW) under drought stress. P1 = Year 2014–15, P2 = Year 2015–16, P3 = Year 2016–17.

A F_11_ RIL population developed by crossing chickpea varieties Pusa 362 (100 seed weight- 16.3 g, yield/plant- 125.67 g, RWC-70.99%, and MSI- 80.99%) and SBD 377 (100 seed weight- 32.39 g, yield/plant- 32 g, RWC- 40.88% and MSI- 53.88%), showed a considerable amount genetic variation for traits under drought stress. The RIL population showed segregation for all the four drought tolerance traits analyzed in this study. The 3 years descriptive statistics for these traits are summarized in Table 2. For all the traits analyzed, a normal frequency distribution curve was generated for three years (Fig. 2) and a relatively higher degree of genetic variation was observed. The CV of RWC was 9.4%, 9.21%, and 9.12% during 2014–15, 2015–16 and 2016–17 respectively. The CV for MSI was 27.99% (2014–15), 27.17% (2015–16), and 26.27% (2016–17). The CV of YLD was 46.57% (2014–15), 46.89% (2015–16), and 46.5% (2016–17). The CV for 100SW was 18.32% (2014–15), 17.45% (2015–16), and 17.1% (2016–17). The Pearson correlations showed the significant correlation among four traits under drought stress to establish relationship between traits under study (Fig. 3).

**Table 2:** Descriptive statistics for the traits analysed in the ‘Pusa 362/ SBD 377’ RIL population of chickpea.

Trait	Pusa 362	SBD 377	Min	Max	Mean	SD	CV
MSI	80.99	53.58	19.62	82.87	53.57	3.77	7.24
RWC	70.99	48.88	48.68	82.78	64.4	5.87	9.02
SWD	16.37	32.39	10.94	35.55	23.95	3.90	6.06
YLD	125.67	32.00	6.34	177.67	64.80	5.77	8.16

**Figure 4:**
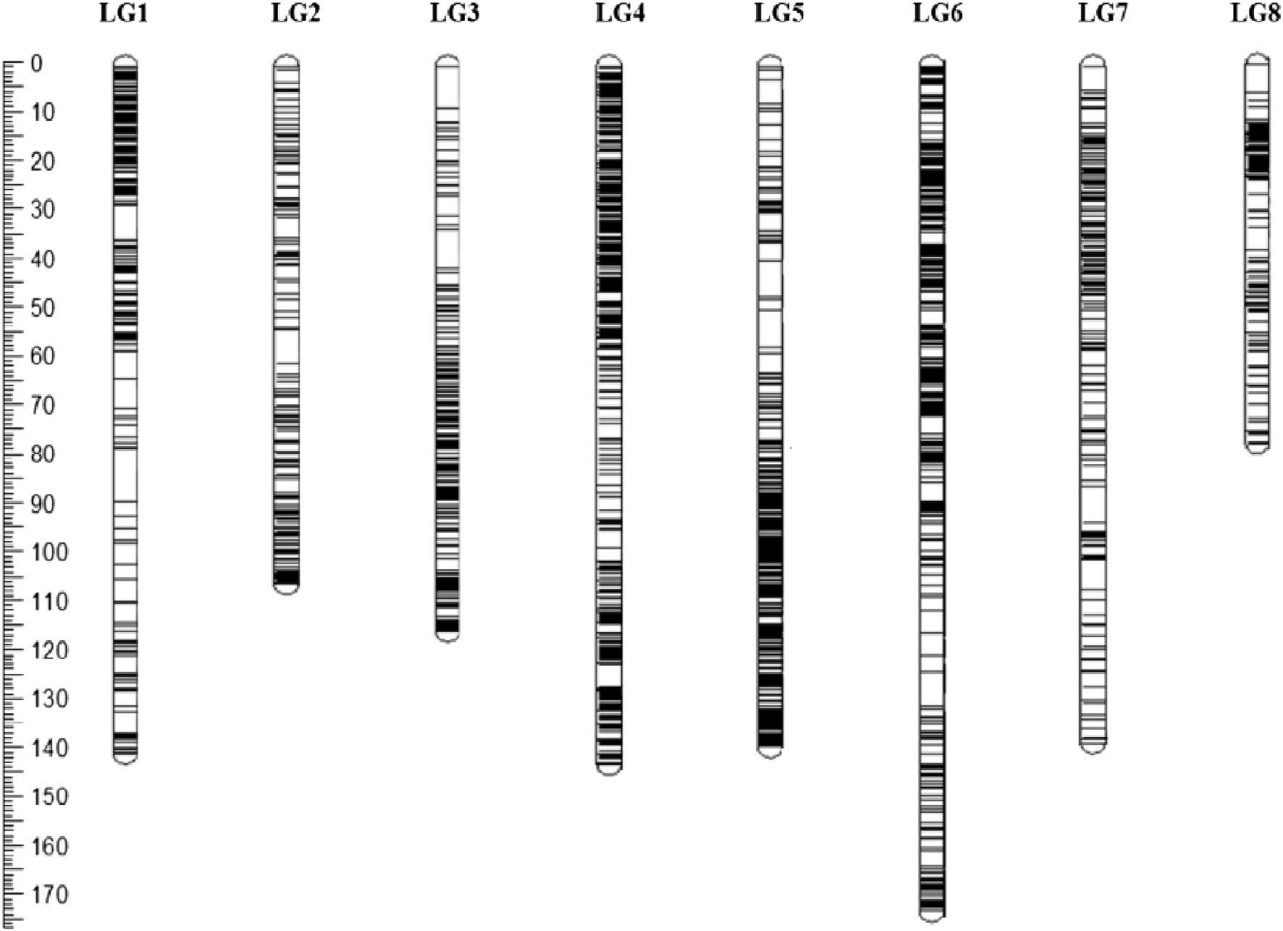
Intra-specific linkage map of chickpea constructed using the RIL population derived from the parental lines Pusa 362 X SBD 377. The scale shown on the left is in cm. The eight chromosomes are shown as vertical bars and each horizontal line on the bar represents a SNP marker. Aggregation on horizontal lines reflects higher marker density on that chromosome.

Genome wide RAD SNPs were utilized to generate a high-density linkage map of the RIL population. A total 3,237 SNPs identified by stringent filtering of data for SNP quality and goodness of fit to the expected 1:1 segregation ratio for RILs were used to develop linkage maps of the eight chickpea chromosomes (Fig. 4, Supplementary Table S4). The linkage groups were numbered CaLG1-CaLG8 according to the earlier published maps^12^**.** The lengths of individual linkage groups ranged from 81.0 cm for LG8) to 178.1 cm for LG6 and the total map length of the eight linkage groups was 1069.1 cm. The number of SNPs mapped per linkage group varied from 128 (LG8) to 636 (LG6) followed closely by LG4 which had 633 mapped SNPs. The average marker interval for genome was 0.33 cm with LG4 exhibiting highest density linkage group (0.23 cm) and LG8 having the lowest marker density (0.63 cm). A summary of the marker information for the eight linkage groups of chickpea is presented in Table 1.

**Figure 5:**
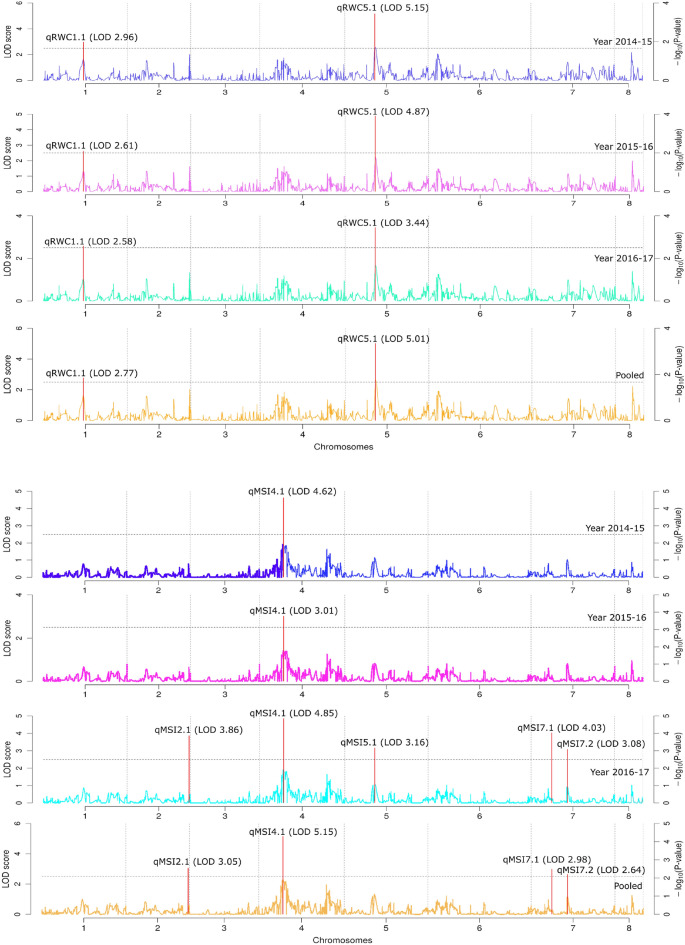

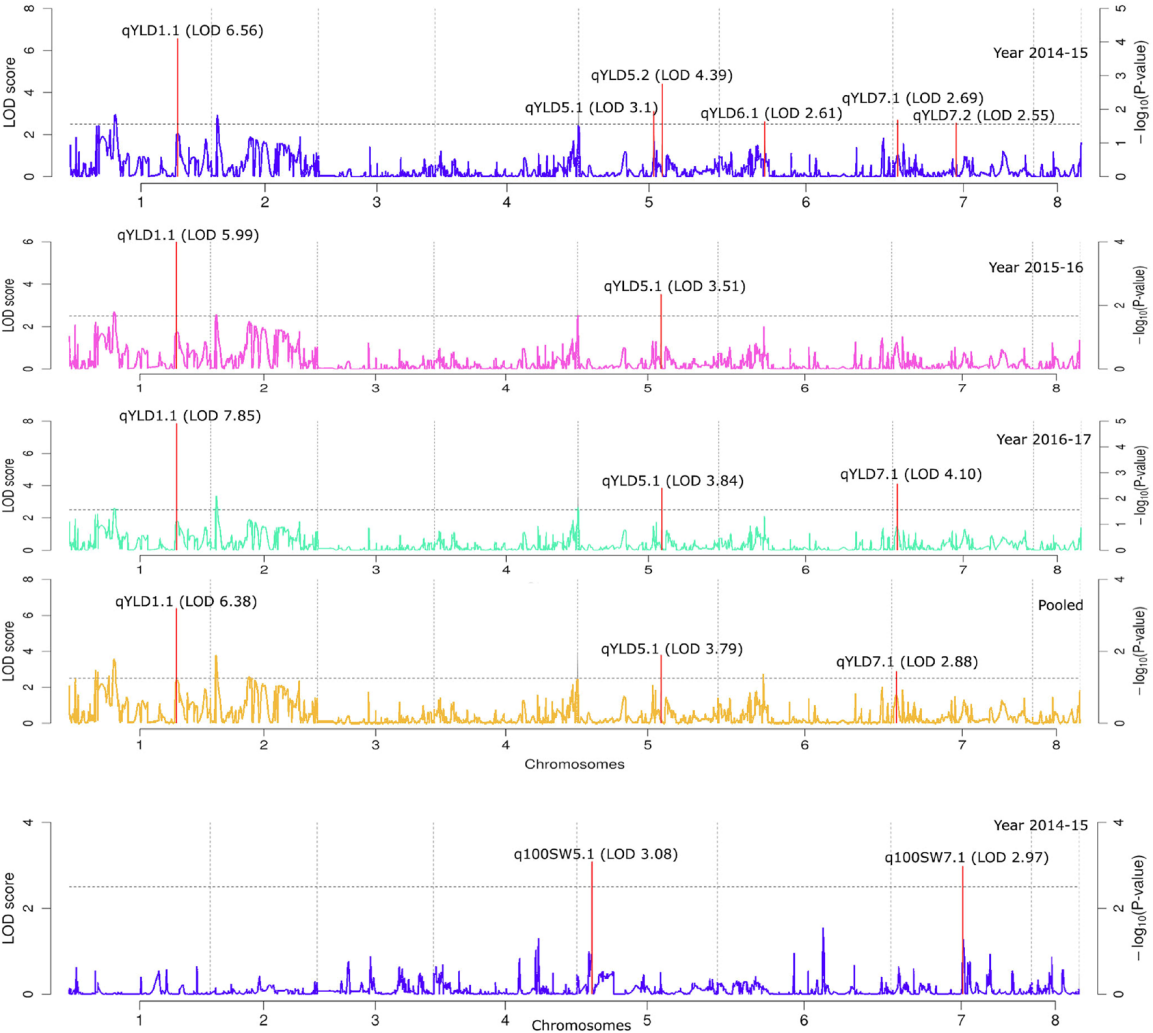
Quantitative trait loci (QTLs) for relative water content (RWC), membrane stability index (MSI), yield per plant (YLD), and seed weight (100SW). The broken lines indicates the genome-wide significance LOD threshold, and the vertical red lines for main QTLs.

We utilized this high-density genetic map constructed using GBS based SNPs to identify QTLs for the four traits, which were distributed on all the linkage groups (LGs) except LG3 and LG8 (Fig. 5, Table 3). The analysis using genome-wide composite interval mapping (GCIM) method identified 11 QTLs associated to RWC, MSI, YLD, and 100SW on five different Linkage Groups (LG 1, 4, 5, 6, and 7) based on year 2014–15 phenotypic results. However, in next years (2015–16), five QTLs have been identified to be linked to the traits RWC, MSI, and YLD on the three different Linkage Groups (LG 1, 4, 5) and 10 QTLs with the traits RWC, MSI, and YLD on five different Linkage Groups (LG 1, 2, 4, 5, 7) in the year 2016–17. All the 22 identified QTLs (LOD ≥ 2.5) represented four different traits located on different LGs of chickpea except LG 3, and LG 8.The QTLs for RWC (*qRWC1.1* and *qRWC5.1*) identified on LG 1 and LG 5 were observed consistently for three years in the same physical position under drought stress (Table 3; Fig. 5). For *qRWC1.1* the LOD values of 2.77 and phenotypic variance of 80.76 were obtained for the trait from the pooled data analysis. Similarly for *qRWC5.1* the LOD value of 5.01 and phenotypic variance of 3.1 were observed from pooled data analysis (Table 3; Fig. 5).The additive effect 15.36 for *qRWC1.1*and 1.47 for *qRWC5.1* was observed form pooled data (Table 3). The position of *qRWC1.1* was at 69 cm with in the marker interval of S1_21926081 and S1_24070908 on LG 1 QTL *qRWC1.1* and peak marker was identified for *qRWC5.1* at 51.97 cm on LG5. The QTLs for MSI were detected on four different linkage groups namely LG2, LG4, LG4, LG5, and LG7. However the QTLs on LG4 (*qMSI4.1*) was found to be consistent across the year and at same physical position in pooled data analysis (Table 3; Fig. 5 ). The QTLs for MSI on LG2 (*qMSI2.1*) LG5 (*qMSI5.1*), and on LG7 (*qMSI7.1* and *qMSI7.2*) were detected in experimental year 2015–16 and in pooled data analysis. The LOD values for these QTLs ranged from 5.15 (*qMSI4.1*) to 2.64 (*qMSI7.2*) in pooled data analysis. These QTLs explained phenotypic variance of 90.68% (*qMSI7.1*), 0.2% (*qMSI7.2*), and 9.12% (*qMSI4.1*), respectively in pooled data analysis**.** The position of *qMSI2.1* was at 107 cm with single peak marker S2_35782996 on LG2, similarly for *qMSI4.1* peak marker was identified at 41.42 cm position on LG4. Other QTLs, *qMSI7.1* and *qMSI7.2* were mapped at 35.61 cm and 62.8 cm position within marker intervals of S7_11812441-S7_11870532 and S7_20579963-S7_21595527 respectively on LG7 (Table 3, Fig. 5). In the case of q100SW, the two QTLs were mapped on two different linkage groups i.e. LGs 5, and 7 in a single experimental year 2014–15. The LOD values ranged from 3.08 (*qSWD5.1*) to 2.97 (*qSWD7.1*). The phenotypic variance explained (PVE) by these two QTLs ranged between 44.15% (*qSWD5.1*) and 49.58% (*qSWD7.1*) (Table 3, Fig. 5).

**Table 3:** Quantitative trait loci (QTLs) associated with the traits: membrane stability index (MSI), relative water content (RWC), seed weight under drought (SWD) and yield under drought (YLD) LG: Linkage Group. Positive additive effect indicates that the trait is contributed by drought tolerant parent Pusa 362, whereas negative additive value indicates contribution by sensitive parent SBD 377.

Year	QTLs	LG	Position (cm)	Additive effect	LOD	LM	RM	PVE (%)	Genomic Region (Mb)
2014–15	*qRWC1.1*	1	69	17.19	2.96	S1_21926081	S1_24070908	83.83	2.14
	*qRWC5.5*	5	51.97	1.51	5.15	S5_17323202	S5_17323202	2.67	–
	*qMSI4.1*	4	41.52	163.3	4.62	S4_13841401	S4_13841401	99.04	–
	*qYLD1.1*	1	110	17.85	6.56	S1_36014093	S1_37647656	–	1.63
	*qYLD5.1*	5	77.09	467.93	3.1	S5_25697492	S5_25697492	100	–
	*qYLD5.2*	5	85.75	2.07	4.39	S5_28583402	S5_28583402	–	–
	*qYLD6.1*	6	46.63	− 128.83	2.61	S6_15544854	S6_15544854	–	–
	*qYLD7.1*	7	4.8	− 26.82	2.69	S7_610723	S7_2360523	–	1.75
	*qYLD7.2*	7	64.85	− 56.99	2.55	S7_21616294	S7_21616294	–	–
	*q100SW5.1*	5	15.41	12.48	3.08	S5_4373523	S5_5404385	44.15	1.03
	*q100SW7.1*	7	73.8	− 12.58	2.97	S7_24190717	S7_25218332	49.58	1.03
2015–16	*qRWC1.1*	1	69	14.08	2.61	S1_21926081	S1_24070908	77.71	2.14
	*qRWC5.1*	5	51.97	1.43	4.87	S5_17323202	S5_17323202	3.55	–
	*qMSI4.1*	4	41.52	157.05	3.01	S4_13841401	S4_13841401	98.99	–
	*qYLD1.1*	1	110	18.11	5.99	S1_36014093	S1_37647656	79.53	1.63
	*qYLD5.2*	5	85.75	− 0.38	3.51	S5_28583402	S5_28583402	5.72	–
2016–17	*qRWC1.1*	1	69	14.83	2.58	S1_21926081	S1_24070908	79.4	2.14
	*qRWC5.1*	5	51.97	1.47	3.44	S5_17323202	S5_17323202	3.15	–
	*qMSI2.1*	2	107.35	377.17	3.86	S2_35782996	S2_35782996	–	–
	*qMSI4.1*	4	41.52	315.18	4.85	S4_13841401	S4_13841401	17.59	–
	*qMSI5.1*	5	51.97	− 0.07	3.16	S5_17323202	S5_17323202	–	–
	*qMSI7.1*	7	35.61	682.12	4.03	S7_11812441	S7_11870532	82.41	0.06
	*qMSI7.2*	7	62.8	134.12	3.08	S7_20579963	S7_21595527	–	1.02
	*qYLD1.1*	1	110	19.22	7.85	S1_36014093	S1_37647656	67.16	1.63
	*qYLD5.2*	5	85.75	− 0.52	3.84	S5_28583402	S5_28583402	6.28	–
	*qYLD7.1*	7	4.8	− 31.04	4.1	S7_610723	S7_2360523	12.12	1.75
Pooled	*qRWC1.1*	1	69	15.36	2.77	S1_21926081	S1_24070908	80.76	2.14
	*qRWC5.1*	5	51.97	1.47	5.01	S5_17323202	S5_17323202	3.1	–
	*qMSI2.1*	2	107.35	260.3	3.05	S2_35782996	S2_35782996	–	–
	*qMSI4.1*	4	41.52	212.84	5.15	S4_13841401	S4_13841401	9.12	–
	*qMSI7.1*	7	35.61	670.91	2.98	S7_11812441	S7_11870532	90.68	0.06
	*qMSI7.2*	7	62.8	314.82	2.64	S7_20579963	S7_21595527	0.2	1.02
	*qYLD1.1*	1	110	19.1	6.38	S1_36014093	S1_37647656	67.81	1.63
	*qYLD5.2*	5	85.75	− 0.59	3.79	S5_28583402	S5_28583402	6.29	–
	*qYLD7.1*	7	4.8	− 30.08	2.88	S7_610723	S7_2360523	11.34	1.75

Three QTLs (*qYLD1.1*, *qYLD5.2,* and *qYLD7.1*) were identified on three different linkage groups including LGs 1, 5, and 7 for the trait yield per plant in pooled data analysis. The LOD values ranged from 6.38 (*qYLD1.1*) to 2.885 (*qYLD7.1*). The phenotypic variance explained by these three mapped QTLs ranged from 67.81% (*qYLD1.1*) to 6.29% (*qYLD5.2*). The pooled data analysis of this result showed that the alleles from parent Pusa 362 favored yield at all the loci except *qYLD5.2* and *qYLD7.1* (Table 3). The flanking regions of the identified SNPs were used to anchor the consistent QTLs to the chickpea physical map. The QTL interval for yield, *qYLD1.1* on chromosome 1 spanning 1.63 Mb region were flanked by markers S1_36014093-S1_37647656at 110 cm position while QTL *qRWC* flanked by S1_21926081 and S1_24070908 spanning 2.14 Mb region. The QTLs *qMSI7.1*, *qMSI7.2* and *qYLD7.1* occupied a physical distance of around 0.06, 1.02, and 1.75 Mb respectively on the chromosome 7 (Fig. 5 Table 3).

### Candidate genes for drought tolerance in the QTL intervals

**Figure 6:**
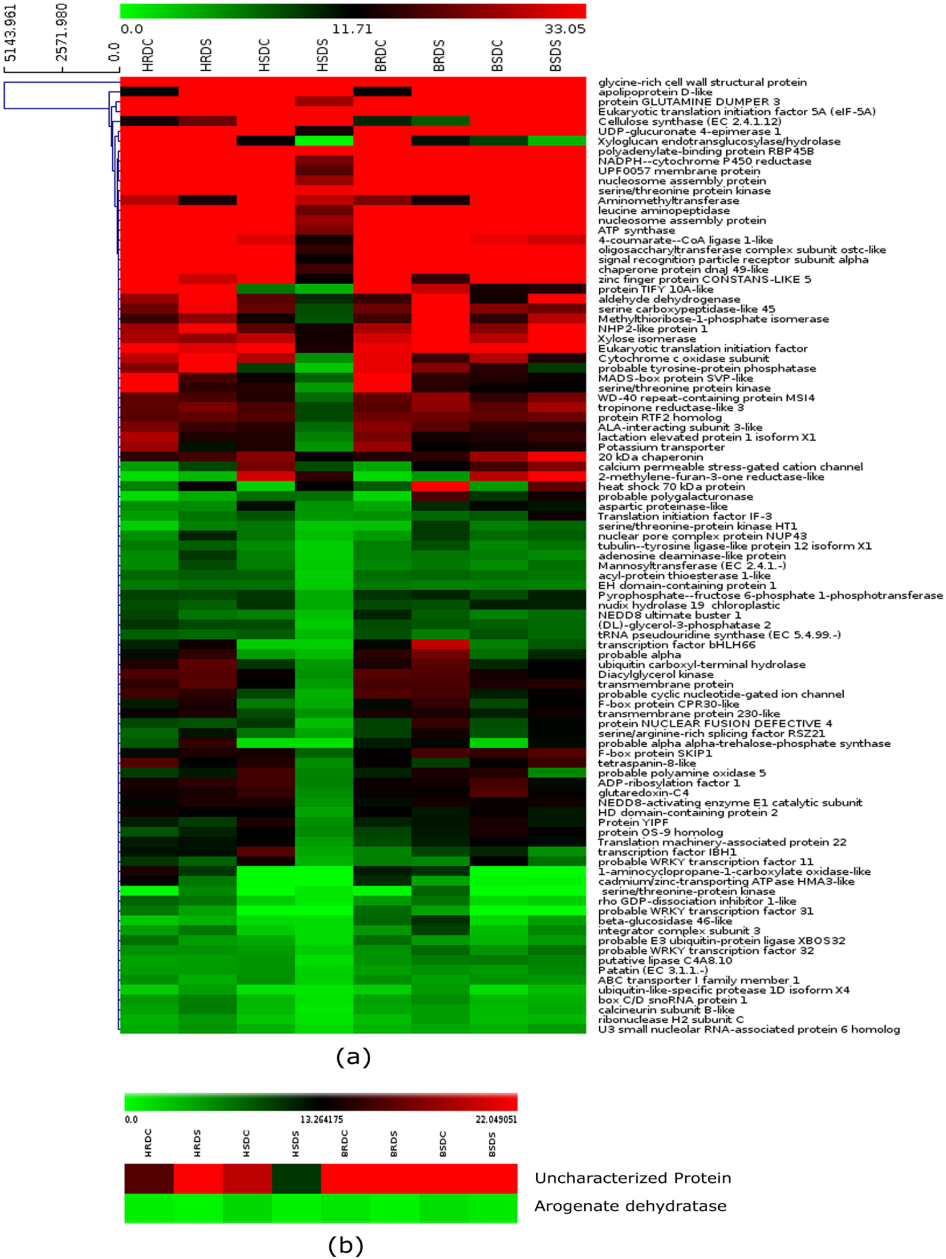
(**a**) Differential expression of selected putative candidate genes in different chickpea root and shoot under control and drought stress conditions. (**b**). Heatmap depicting differentially expressed genes with haplotype identified.Color scale in block represents differences in gene expression; highest (red color) and lowest (green color) gene expression. Hashem root drought control-HRDC, Hashem shoot drought control-HSDC, Hashem root drought stress-HRDS, Hashem shoot drought stress-HSDS. Bivanij root drought control-BRDC, Bivanij shoot drought control-BSDC, Bivanij root drought stress -BRDS, Bivanij shoot drought stress-BSDS.

The 9 consistent QTLs mapped in this study spanning total of 6.6 Mb region on five chickpea chromosomes were analysed for the identification of candidate genes for membrane stability complex (MSI), relative water content (RWC), and yield per plant (YLD). A total number of 369 genes were identified in the 6.6 Mb regions (Supplementary Table S5). *In-silico* expression profiling was done for all 369 genes using the available transcriptome data on root and shoot tissues from the chickpea varieties, Hashem (drought sensitive) and Bivanij (drought tolerant). Out of 369 genes, 326 genes were differentially expressed under drought conditions. Among these 369 genes, 37 genes were identified for *qMSI*, 80 genes for *qRWC*, and 252 genes for *qYLD* QTL (Fig. 6, Supplementary Table S5). These candidate genes included genes coding for proteins such as DEAD box ATP dependent RNA helicase, chaperonins, sugar transport proteins, E3 ubiquitin protein ligase, heat shock 70 kDa proteins, aquaporins, amino acid permeases, UDP glycosyl transferase, ABC transporter proteins, MADS-box protein, etc. Genes coding for transcription factors (TFs) like ethylene-responsive transcription factor, MYB, zinc finger protein, WRKY were also found to have drought specific expression. Gene ontology (GO) was done to identify characteristic biological attributes of in silico RNA-Seq. data. The GO studies revealed that cell redox homeostasis, DNA conformation change, protein modification process, regulation of transcription were enriched under drought stress conditions. The identification of these enriched terms holds tremendous potential in expanding our comprehension of the precise impact of differentially expressed genes within the context of drought stress, and thereby offering valuable insight for further investigation and understanding. Therefore, these genes are putative candidate genes in the mapped QTL regions, which require further validation by genetic fine mapping and transformation studies. To investigate the status of differentially expressed genes in different class of pathways, gene expression information was mapped to the KEGG pathways. The KEGG analysis was done to bring down the number of candidate genes. This reduced the candidate genes from 369 to 99(Supplementary Table S5). Pathways analysis and functional annotation showed that differentially expressed genes were clustered in several signaling pathways such as Arginin and proline metabolism, cellular senescence, biosynthesis of secondary metabolites, ubiquitin mediated proteolysis, pyruvate metabolism, MAPK signaling pathways, glutathione metabolism (Supplementary Table S5)**.**

To validate the identity of the mapped QTLs as well as the candidate genes mined, we undertook the haplotype analysis. We first mined the number of genes harboring SNPs within the QTL intervals and identified 5 out of 9 consistent QTLs which harbored genic SNPs. Two QTLs with maximum number of SNPs, *qRWC1.1* (10 SNPs) and *qYLD7.1* (121 SNPs) were selected for haplotype analysis. In the *qRWC1.1* region, six candidate genes were found of which only one gene, LOC101514724, encoding ATP-dependent DNA helicase Q-like 3, was associated with more than two SNPs. However, no significant difference was found in RWC between the two haplotypes of *qRWC1.1* (64.7% vs 66.5%). Of the 74 candidate genes in *qYLD7.1*, four candidate genes, LOC101498779, LOC101501730, LOC101514620 and LOC101494835 harboured more than one SNP and hence were selected for haplotype analysis. Two haplotypes each, identified in LOC101494835 and LOC101498779, showed a significant difference in yield (65.31 g vs 89.51 g and 65.61 g vs 89.99 g grams). The haplotype GGTC/TACT were associated with loci S7_993031, S7_994976, S7_995674, and S7_995691 in LOC101498779, which encodes arogenate dehydratase/prephenate dehydratase 1 and KEGG pathways analysis showed that this gene came under metabolic pathways including biosynthesis of secondary metabolites and amino acids phenylalanine. Similarly, the haplotype CGAA/TTTG was associated with loci S7_2228317, S7_2228318, S7_2228372m, and S7_2230434 in LOC101494835 gene, whose orthologs in *Glycine max* encodes MHD domain-containing proteins that are associated with flavonoid biosynthesis. This gene was observed to be differentially expressed between the drought tolerant and drought sensitive varieties of chickpea (Fig. 6B) providing transcriptome level evidence of the significance of this gene under drought stress in chickpea.

## Discussion

### Efficiency of GBS based SNPs in QTLs mapping

Terminal drought that is, shortage of water during the reproductive phase is the most detrimental form of drought stress, leading to a huge yield losses in agricultural crops (45–69%)^6^. In order to devise breeding strategies for the development of drought tolerant chickpea varieties, there is an urgent need to identify novel QTL/genes for drought stress tolerance. A high-density, saturated genetic map can provide high-resolution molecular markers linked with the genomic regions controlling the complex drought tolerance traits for utilization in marker-assisted breeding. Deployment of molecular breeding for development of drought resilient superior chickpea cultivar, it is pre-requisite to unravel the genetic basis of drought tolerance and identification of tightly linked molecular marker. Most of the early molecular linkage maps of chickpea were constructed by using SSR markers with marker low-density, a problem further compounded by low level of inter-varietal polymorphism due to narrow genetic base of the crop^21–23^. The large QTL intervals mapped using SSR markers, may lead to introgression of undesirable linkage drag during MAS. SNP markers overcome this limitation due to their higher abundance in the genome. High-density chip arrays and GBS platforms allow simultaneous identification and genotyping of thousands of polymorphic SNPs for creating high-density linkage maps and QTL mapping. In chickpea, SNP markers have been used in recent past for mapping QTLs for drought tolerance, seed traits, *Ascochyta* blight, Fe and Zn content, pod borer resistance and yield traits^12,17,24–29^.

In the present study, GBS approach was used for high throughput SNP genotyping of a RIL population derived from cross between two *desi* chickpea varieties, Pusa 362 (tolerant) and SBD 377 (sensitive), with contrasting values for four drought tolerance parameters, namely MSI, RWC, SW and YLD under drought stress. The traits were selected keeping in view the impact of reproductive stage drought stress on drastic reduction of grain yield. MSI and RWC are physiological traits which measure the cellular response to drought stress and are considered as reliable parameters for quantifying the plant drought stress tolerance. SWD and YLD under drought stress are a direct measure of drought tolerance. Two parents (Pusa 362 and SBD 377) were selected due to their contrasting phenotypic traits associated with drought tolerance (Table 2). Pusa 362 exhibits superior drought tolerance traits when compared to SBD 377. This can be observed in its higher Relative Water Content (RWC) of 70.99% compared to 40.88% in SBD 377, suggesting that Pusa 362 retains more water under drought conditions. Moreover, Pusa 362 has a Membrane Stability Index (MSI) of 80.99% indicating greater cell membrane stability under drought stress than SBD 377, which has an MSI of 53.88%. Yield differences are also evident between the two parental lines. Pusa 362 has a yield per plant of 125.67 g, which is almost four times higher than that of SBD 377, which stands at 32 g. This demonstrates the superior productivity of Pusa 362 under the same conditions. Seed weight contrasts between the two varieties are evident as well, with Pusa 362 having a 100-seed weight of 16.3 g, while SBD 377 has almost double that at 32.39 g. These differences in drought tolerance and yield traits between the two parental lines led to the observed genetic variation in their F11 RIL population offspring. The contrasting traits of the parents serve as a foundation to better understand the phenotypic range and variation present in the RILs, providing valuable insights into the genetic determinants of drought tolerance in chickpea. While the QTLs controlling RWC and MSI have been mapped in several crops including rice, wheat and maize, and recently the QTLs for cell membrane stability traits for heat stress have reported in chickpea^30^. The F_11_ RIL population developed and used in this study exhibited large variation for these traits with normal frequency distribution indicating quantitative inheritance over three consecutive years. In present study, the presence of high coefficients of variation (CV) for YLD (> 46%) across the consecutive three years within the F11 RIL population have observed. By the F11 generation, generally most loci in recombinant inbred lines (RILs) should be homozygous, effectively fixing most genetic variability^31^. However, despite these expectations, we observed a notable high CV, suggesting the influence of various intertwined factors. Residual heterozygosity, although expected to be minimal, could still persist, leading to some ongoing genetic segregation and, consequently, variability. Epistatic interactions are also important contributors, given that yield is a multifaceted trait, and interactions between various QTLs can manifest in broader phenotypic variability than individual QTL effects alone^32^^**.**^ Additionally, while controlled environments, such as those under rainout shelters, minimize variability, micro-environmental fluctuations like subtle changes in temperature or humidity can still impact plant yield^33^. It's imperative to also consider that even in such controlled settings, the genetic makeup of plants can interact in complex ways with these subtle environmental nuances, resulting in gene-by-environment (GxE) interactions and the observed variability^34^. This interplay of genetic and environmental factors in our F11 RIL population underscores the challenges of interpreting high CV in legume yield studies, even under ostensibly controlled conditions. Significant positive correlations of MSI, RWC and SWD with grain yield under drought stress, suggesting that these are genuine parameters for assessing drought tolerance. Positive correlation between 100 seed weight and grain yield under drought stress were observed and it has also been reported earlier in chickpea^35^. It indicates that yield related traits could be vital parameters and primary objective for developing drought tolerant crop plants and legumes including chickpeas. The traits targeted for improving drought tolerance must have strong correlation with grain yield under drought stress conditions^36^.

### Elucidation and genotyping of genome wide SNPs and linkage map construction

A total of 35,502 SNPs were identified between Pusa 362 and SBD 377 by alignment of Illumina sequence reads to the chickpea reference genome^15^, but only 3237 (9%) of these were suitable for linkage mapping, after filtering for missing values and segregation distortion. This underlines the limitation of GBS in finding commonly genotyped SNPs across individuals due to random Poison distribution of the sequence reads in the genome of individual samples. This can be improved by increasing the fold coverage of sequence data which adds to the sequencing and informatics costs. Hence, recently there has been a shift towards use of high-density chip array-based SNP genotyping for QTL mapping in crop plants including chickpeas^17,37,38^. The highest number of SNPs were mapped to chromosome 6 (636), followed closely by chromosome 4 (633). These account for 19% of the SNP markers mapped. The mapping of higher number of SNPs on the 4th chromosome (CaLG4) has previously been reported by Jaganathan et al. 2015^24^ and has been attributed to the possible presence of repeat rich regions on the chromosome. About 51% of the SNPs were mapped to the genic regions of the genome with 23% of intronic SNPs. 22% of the genic SNPs mapped to the exons in the genome. Among the genes which carried highest number of exonic SNPs were the genes coding for pentatricopeptide repeat-containing proteins. These proteins have been shown to be involved in regulating plant responses to drought, salinity and cold^39^. The presence of large number of exonic SNPs in these genes might be indicative of their role in drought stress tolerance in chickpea. The average marker density across the chromosomes was 0.33 cm, which is similar to chickpea SNP maps reported by Verma et al. 2015^12^(3,228 SNPs spanning 1006.98 cm), Barmukh et al. 2020, 2021^28,29^(3,873 SNP loci spanning 949.27 cm and 3,818 SNP loci spanning 1064.14 cm), denser than inter and intra-specific maps reported by Gaur et al. 2012^40^ (1063 markers,1.7 cm)**,** Hiremath et al. 2012^41^ (1328 markers, 0.59 cm), Deokar et al. 2014^42^ (1336 markers, 0.59 cm), but lower density than chip array-based linkage map reported by^17^ (13 679 SNPs spanning 1033.67 cm and 7769 SNPs spanning 1076.35 cm).

### High-Resolution QTL mapping

Utilization of RIL population with high density genetic map and phenotyping under rainout shelter allowed to precise mapping of genomic region/s for drought stress in chickpea. A positive and significant correlation between the yield and yield related traits like seed weight could be an important parameter for developing drought resilient chickpea genotypes, which indicated that yield traits have considered to be the important objective for improving drought tolerance in agriculturally important crops including chickpea. We have identified 9 consistent QTLs for RWC (*qRWC1.1, qRWC5.1*), MSI (*qMSI2.1, qMSI4.1, qMSI7.1, and qMSI7.2*), and YLD (*qYLD1.1, qYLD5.2, and qYLD7.1*) which may be used for marker assisted breeding for drought tolerance in chickpea. In addition to mapping of QTLs for yield under drought stress environment, to the best of our knowledge, this is the first study that reports mapping of QTLs for physiological traits like MSI and RWC. Four QTLs for MSI, namely *qMSI2.1, qMSI4.1, qMSI7.1, and qMSI7.2* with PVE range from 90.68% (*qMSI7.1*) to 0.2% (*qMSI7.*2) were found on linkage groups LG 2, LG 4 and LG 7, respectively, while the two consistent QTLs for RWC viz*.*, *qRWC1.1* (PVE- 80.76), and *qRWC5.1* (PVE- 3.1), were identified on LG 1, and 5 respectively. Three major genomic regions spanning three robust QTLs (PVE: 67.81–6.29%) associated with yield per plant agronomic traits (YLD) were identified and mapped on three different Linkage Groups (LG 1, LG 5, and LG 7) of high density genetic map of chickpea. On the basis of physical position of SNPs marker covering the respective QTL intervals, the reliability of mapped QTLs for YLD were determined by comparing their underlying genomic region with earlier studies on genomic mapping^10,25,43,44^**.** In contrast to previous studies the QTLs identified in this study, especially the QTLs for yield were observed to overlap with previously mapped QTLs^10,25^. All these nine mapped QTLs reported by our study can be considered as robust QTLs showing phenotypic expression and have major effects except *qRWC5.1* (PVE 3.1%), *qMSI4.1* (PVE 9.12%), *qMSI7.2* (PVE 0.2%), and *qYLD5.2* (PVE 6.29%) on traits individually with PVE > 10% each. The consistent QTLs across different studies, can be of immense utility for pyramiding for reproductive stage drought tolerance in chickpea.

### Candidate gene analysis associated with mapped QTLs

To identify the candidate genes in the QTL intervals, the genetic map developed in the present study was integrated with available genome and transcriptome resources of chickpea. The *kabuli* chickpea (CDF Frontier) genome^15^ was used for this analysis, because it is better assembled than the *desi* chickpea (ICC 4958) genome^14^ and therefore the possibility of locating the marker intervals on the chromosomes is higher. A total of 326 putative candidate genes showed differential gene expression. These genes encoded proteins associated with different metabolic processes and pathways such as biosynthetic pathways, signaling pathways, photosynthesis, etc. A large majority of these genes are involved in the biosynthesis of osmolytes like amino acids, polyamines and sugar alcohols. These genes have been shown to express under abiotic stress conditions as adaptive features to maintain plant water potential^45,46^. Similarly, the genes associated with photosynthetic, starch biosynthesis and UDP-glucose biosynthesis pathways, have been shown to be induced under drought stress in chickpea^46^. The synthesis of UDP-Glucose is a pre-requisite for imparting mechanical strength and cell-wall remodeling to protect plant from abiotic stress^47^. Genes coding for different TF families were identified and were differentially expressed under drought stress conditions. These are also involved in hormonal signaling, in response to hormones like abscisic acid, auxin, gibberellin and cytokinin, indicating a vital role of plant hormones and their cross-talk in drought stress^48–51^.Utilizing and predictive annotation approaches to identify candidate genes located at loci harboring SNPs has the potential to serve as a highly effective strategy in pinpointing causal genes^52^. To ascertain the veracity of the mapped QTLs and the potential candidate genes involved, haplotype analysis was performed, which revealed the presence of 5 QTLs among the 9 initially identified QTLs. From these, 2 QTLs, namely *qRWC1.1* and *qYLD7.1,* were selected due to their high SNPs density within the QTL regions. While the candidate gene based haplotype analysis could not support the findings of *qRWC1.1*, two candidate genes in the *qYLD7.1* displayed two distinct haplotypes that exhibited a substantial phenotypic differences. These two haplotypes could be associated with metabolic pathways for secondary metabolites biosynthesis including amino acid and flavonoid biosynthesis as found from the KEGG and GO analyses. Arogenate Dehydratase/Prephenate Dehydratase 1 (LOC101498779) gene encodes an enzyme involved in the terminal steps of phenylalanine biosynthesis. Phenylalanine is not just an essential amino acid; it is also a precursor to many secondary metabolites, including flavonoids, lignins, and alkaloids. Phenylalanine-derived compounds can play protective roles under stress. Lignins, for example, can contribute to cell wall rigidity and reduce water loss^53^. The exact function of MHD Domain-Containing Proteins (LOC101494835) in plant might be uncertain, the orthologs in Glycine max are associated with flavonoid biosynthesis. Flavonoids are versatile secondary metabolites playing roles in UV protection, signaling, and stress responses. Flavonoids antioxidant properties make them crucial in scavenging reactive oxygen species, which increase under stress conditions like drought. They can protect cellular structures from oxidative damage and maintain cell homeostasis^54^. Flavonoids have an antioxidant properties and have the ability to eliminate reactive oxygen species (ROS) in response to drought stress^55^. By impeding the metabolic functions of enzymes involved in ROS production pathways flavonoids induce activation of the antioxidant defense system. Given that phenylalanine is a precursor to flavonoids, these two genes might be part of a coordinated response to drought. Enhanced phenylalanine production could fuel flavonoid biosynthesis, boosting the plant's antioxidant capacity during drought^56^**.**These identified genes could impact other stress responses. For instance, flavonoids also play a role in UV protection, pathogen defense, and allelopathy. An upregulated flavonoid pathway could enhance multiple stress defenses simultaneously. Phenylalanine-derived compounds like lignins can also play roles in pathogen defense and structural integrity^57^.The two identified candidate genes play roles in interconnected pathways pivotal for plant stress responses. Beyond their direct metabolic functions, they contribute to a broader network of responses that can enhance plant resilience not just to drought but potentially other stresses as well. This holistic understanding underscores their significance and potential as targets for crop improvement. The QTL regions associated with these genes, whose role in drought stress has been well known, strongly support the hypothesis for association of the identified QTLs to drought stress tolerance.

## Conclusions

The high-density linkage map developed in this study is likely to be of immense utility for high resolution mapping of QTLs for drought tolerance traits in chickpea. This enabled identification of closely linked SNP markers, which can be used for the introgression of superior alleles of the genes for drought tolerance traits through MAS for developing drought tolerant cultivars. In this study we identified novel QTLs for physiological traits, membrane stability index (MSI), relative water content (RWC), and yield/plant (YLD) under drought stress. The QTLs for membrane stability index (MSI) and relative water content (RWC), have been mapped for the first time in chickpea. To validate the accuracy of the mapped QTLs and determine the potential candidate genes implicated, a comprehensive haplotype analysis was conducted. The analysis unveiled the existence of five QTLs out of the initial nine QTLs that were identified. The identification of differentially expressed candidate genes in these QTL regions, paves way for better understanding of the biological pathways and molecular mechanisms involved in drought stress response with ultimate aim of introgressing these genomic regions, using MABC, for developing superior drought tolerant chickpea genotypes.

## Materials and methods

### Plant material and genomic DNA extraction

An F_11_ RIL population developed by crossing chickpea varieties Pusa 362 (drought tolerant, small seeded) and SBD 377 (drought sensitive, bold seeded) comprising of 186 individuals were used to identification of genomic regions and candidate genes for drought tolerance. Fresh young leaves were used for genomic DNA isolation using the CTAB method with minor modifications^58^. The isolated DNA was checked for quality by electrophoresis in 0.8% agarose gel and quantified using Nanodrop 8000 spectrophotometer equipment (Thermo-Scientific).

### Phenotyping for drought tolerance parameters and statistical analysis

The set of 186 RILs along with the parents Pusa 362 and SBD 377 were grown for phenotyping in augmented block design for three consecutive years during the *rabi* season (2014–15, 2015–16 and 2016–17) under rainout shelter conditions at the ICAR-IIPR, Kanpur, Uttar Pradesh, India (26° 26′ 59.7228’’N, and 80° 19′ 54.7356’’E). Each experimental block consisted of a single 1 m plot with a spacing of 0.3 m between rows. In chickpea reproductive phase (particularly flowering and pod formation) is consider as a key step to evaluate drought traits. Exposure to water deficit at reproductive stage may cause yield loss through flower and pod abortion and is consider as a simple and effective screening techniques for terminal drought. Drought stress was imposed by withholding irrigation at the pod formation stage after 90 days after sowing. The parental lines and RILs were phenotyped for four drought tolerance parameters: (i) membrane stability index (MSI), (ii) relative water content (RWC), (ii) seed weight under drought (SWD) and yield /plant (YLD) under drought stress, over three consecutive years from 2014 to 2017. For determination of RWC, the third fully expanded leaf from top of the plants was collected before noon. The leaves were weighed to record the fresh weight (FW), then immersed in double distilled water in Petri plates for 4 h and weighed again to record turgid weight (TW). The samples were then dried at 65 °C for 48 h and weighed to record the dry weights (DW). The data was recorded in triplicates for each of the RILs and parental genotypes. The RWC (%) was calculated using the following formula:

$$ {\text{RWC }}\left( \% \right) \, = \, \left( {{\text{FW}} - {\text{ DW}}} \right) \, / \, \left( {{\text{TW}} - {\text{ DW}}} \right) \, \times { 1}00 $$

To measure the MSI, leaf tissue (0.5 g) was collected in glass vials containing 10 ml of double distilled water. These samples were incubated in a water bath at 40 °C for 30 min. After cooling the tubes to room temperature, electrical conductivity (C1) of the solution was recorded with a conductivity meter (Sanco, India). The tubes were then kept in a boiling water (100 °C) bath for 10 min and electrical conductivity measured (C2), after the tubes attained the room temperature (25 °C). The membrane stability index (MSI) using the following formula:

$$ {\text{MSI }}\left( \% \right) \, = \, \left[ {{1} - \, \left( {{\text{C1}}/{\text{C2}}} \right)} \right] \, \times { 1}00 $$

The seed and yield traits were recorded after threshing and cleaning of seeds. Average 100 seed weight under drought (SWD) was calculated by weighing 100 mature seeds from 10 representative plants of each RIL and the parental genotypes at a seed moisture content of 10%. Average seed yield per plant was recorded from the seed weights of five plants for each RIL and parental genotypes. Statistical analysis to compute mean, standard deviation (SD), coefficient of variation (CV), correlation coefficients, for the traits was performed by using XLSTAT/SPSS v17.0.

### Illumina sequencing and SNP allele calling

To prepare libraries for GBS analysis, genomic DNA from the parental genotypes (Pusa 362 X SBD 377) and 186 RILs was subjected to restriction digestion with a type II restriction endonuclease *Ape*KI according to the protocol of Elshire et al.,^13^. All libraries were pooled and single end sequencing of the fragments was performed using Illumina TrueSeq V3.0 sequencing chemistry, with a read length of 100 bp. The raw sequence reads obtained in the FASTQ file format were processed by filtering through GBS analysis pipeline implemented in TASSEL v3.0^59^. The reads were filtered for the criterion including the presence of perfectly matched barcodes with the four bps remnant sequences of the RE site and a minimum Phred score of 10 across the first 72 bases. The reads were sorted, de-multiplexed and further trimmed to 64 bases for analysis. The filtered reads were mapped to the reference chickpea genome^15^ using Burrows-Wheeler alignment software (BWA). The missing data were imputed using Beagle v3.3.2^60^**,** while retaining the default parameters. The perfectly aligned sequences were used for SNP calling with a with a minor allele frequency (MAF) ≥ 0.2, minimum sequence read depth: 10X and SNP base quality ≥ 20. The distribution of the identified SNPs was visualized across the 8 linkage groups (LGs) using Circos v0.61^61^.

### Structural and functional annotation of the SNPs

The distribution of SNPs in different genomic regions, namely intergenic, exonic, intronic and UTR, was determined to analyse their functional or structural significance. Gene ontology (GO) analysis was performed for the genes containing SNPs to classify them into three principal GO categories, using in-house custom perl scripts and SNP effect predictor (snpEff c3.1 h)^62^. The Benjamini and Hochberg false discovery rate (FDR) correction at a significance level of 5% was done using the BiINGO plugin of Cytoscape v2.6^63^. To identify SNPs present in the transcription factors (TFs) family genes, the peptide sequences of different TF belonging to five leguminous crops (*C. arietinum, Glycine max, Cajanus cajan, Lotus japonicas and Medicago truncatula*) were retrieved from the Plant Transcription Factor database. These sequences were utilized to build HMM profiles for all TF families and categorize the genes with SNPs into different TF families.

### Linkage map construction and QTL mapping

The genome wide SNPs derived from the GBS data on Pusa 362/ SBD 377 RILs were analyzed for segregation distortion against the expected 1:1 ratio using χ^2^-test (*p* < 0.05). Markers showing segregation distortion were excluded from the analysis. The linkage map was constructed using JOINMAP 4.1 program^64^**.** Ordering of the markers was done using regression mapping algorithm RECORD (REcombination Counting and ORDering)^65^. The Kosambi function was used to estimate the map distance between markers^66^ and MapChart^67^ was used for visualization the genetic map. The QTLs associated with the traits under consideration were mapped by integrating the genotypic and phenotypic data on the 186 RILs using QTL.gCIMapping^68^. The default settings were used with a walk speed of 1 cm, window size of 10 cm and a minimum LOD threshold score of 2.5 calculated at 10,000 permutations and significance level, (*P* ≤ 0.05) were used to identify significant QTLs for the traits. The phenotypic variance explained (PVE %) and the additive effect explained by the QTLs were also determined.

### Identification of candidate genes in the QTL intervals

The genomic sequence between the SNP loci flanking the identified QTL intervals were retrieved by BLAST search against the reference chickpea genome^15^ sequence to identify the putative candidate genes controlling the traits The identified sequences were used for gene prediction using FGENESH^69^. *In-silico* expression analysis was performed for the identified candidate genes using available transcriptome data from the root and shoot tissues of chickpea genotypes ‘Bivanij’ (drought tolerant) and ‘Hashem’ (drought sensitive), exposed to drought stress (root-SRX3087500, SRX3087499, SRX3087504, SRX3087503; shoot-SRX3087498, SRX3087497,SRX3087502, SRX3087501)^70^. The transcriptome data was retrieved from NCBI and mapped to the putative candidate genes with the help of the 454 Roche gsMapper (Newblerv2.3.5). A heatmap was generated using the FPKM (Fragments per kilobase per million mapped reads) values to depict the differential gene expression by using the TIGR Multiple Experiment Viewer (MEV) software^71,72^. Biological significance of differentially expressed genes was explored by GO term enrichment analysis including biological processes, molecular functions and cellular components using Blast2GO ^73^. The KEGG enrichment was done using David v6.8. Haplotype analysis for selected genes were done using Haploview ^74^^**.**^

### Ethical approval

The authors declare that the experimental research work involving the growth of plants in this study, was conducted in compliance with relevant institutional, and national guidelines and legislation.

### Supplementary Information

Supplementary Figures.

Supplementary Tables.

## Data Availability

All GBS data has been made available at NCBI under BioProject ID: PRJNA675147. Publically available data sets under BioProject ID: PRJNA396819 were utilized for digital gene expression analysis.

## References

[1] Kaloki, P., Devasirvatham, V., & K.Y. Tan, D. Chickpea Abiotic Stresses: Combating Drought, Heat and Cold, in Abiotic and Biotic Stress in Plants. London, United Kingdom: https://www.intechopen.com/chapters/65127. doi:10.5772/intechopen.83404 (2019).

[2] FAOSTAT. (2020). Available at: http://faostat3.fao.org/home/index.html (accessed17th June 2022).

[3] Hennesy K, Whetton P, Preston B, Stokes C, Howden M (2010). Climate projection. Adapting Agriculture to Climate Change Preparing Australian Agriculture, Forestry and Fisheries for the Future.

[4] Foyer CH (2016). Neglecting legumes has compromised human health and sustainable food production. Nat. Plant..

[5] Purushothaman R, Krishnamurthy L, Upadhyaya HD, Vadez V, Varshney RK (2016). Shoot traits and their relevance in terminal drought tolerance of chickpea (*Cicer arietinum* L.). Field Crop Res..

[6] Nadeem M, Li J, Yahya M, Sher A, Ma C, Wang X, Qiu L (2019). Research progress and perspective on drought stress in legumes: A review. Int. J. Mol. Sci..

[7] Sivasakthi K (2018). Plant vigour QTLs co-map with an earlier reported QTL hotspot for drought tolerance while water saving QTLs map in other regions of the chickpea genome. BMC Plant Biol..

[8] Rehman AU (2011). Mapping QTL associated with traits affecting grain yield in chickpea (*Cicer arietinum* L.) under terminal drought stress. Crop Sci..

[9] Hamwieh A, Imtiaz M, Malhotra RS (2013). Multi-environment QTL analyses for drought-related traits in a recombinant inbred population of chickpea (*Cicer arientinum* L.). Theor. Appl. Genet..

[10] Varshney RK (2014). Genetic dissection of drought tolerance in chickpea (*Cicer arietinum* L.). Theor. Appl. Genet..

[11] Kale SM (2015). Prioritization of candidate genes in “QTL-hotspot” region for drought tolerance in chickpea (*Cicer arietinum* L.). Sci. Rep..

[12] Verma S (2015). High-density linkage map construction and mapping of seed trait QTLs in chickpea (*Cicer arietinum* L.) using genotyping-by-Sequencing (GBS). Sci. Rep..

[13] Elshire RJ (2011). A robust, simple genotyping-by-sequencing (GBS) approach for high diversity species. PLoS One.

[14] Jain M (2013). A draft genome sequence of the pulse crop chickpea (*Cicer arietinum* L.). Plant J..

[15] Varshney RK (2013). Draft genome sequence of chickpea (*Cicer arietinum* L.) provides a resource for trait improvement. Nat. Biotechnol..

[16] Roorkiwal M (2013). Single nucleotide polymorphism genotyping for breeding and genetics applications in chickpea and pigeonpea using the beadxpress platform. Plant Genome.

[17] Roorkiwal M (2018). Development and evaluation of high-density Axiom® CicerSNP Array for high-resolution genetic mapping and breeding applications in chickpea. Plant Biotechnol. J..

[18] Singh S (2020). A 62K genic-SNP chip array for genetic studies and breeding applications in pigeonpea (*Cajanus cajan* L. Millsp.). Sci. Rep..

[19] Deschamps S, Llaca V, May GD (2012). Genotyping-by-sequencing in plants. Biology.

[20] Poland JA, Brown PJ, Sorrells ME, Jannink JL (2012). Development of high-density genetic maps for barley and wheat using a novel two-enzyme genotyping-by-sequencing approach. PLoS One.

[21] Nayak SN (2010). Integration of novel SSR and gene-based SNP marker loci in the chickpea genetic map and establishment of new anchor points with Medicago truncatula genome. Theor. Appl. Genet..

[22] Gaur R (2011). Advancing the STMS genomic resources for defining new locations on the intraspecific genetic linkage map of chickpea (*Cicer arietinum* L.). BMC Genomics.

[23] Choudhary S, Gaur R, Gupta S, Bhatia S (2012). EST-derived genic molecular markers: Development and utilization for generating an advanced transcript map of chickpea. Theor. Appl. Genet..

[24] Jaganathan D (2015). Genotyping-by-sequencing based intra-specific genetic map refines a ‘‘QTL-hotspot” region for drought tolerance in chickpea. Mol. Genet. Genomics.

[25] Kujur A (2015). Ultra-high density intra-specific genetic linkage maps accelerate identification of functionally relevant molecular tags governing important agronomic traits in chickpea. Sci. Rep..

[26] Deokar A, Sagi M, Tar’an B (2019). Genome-wide SNP discovery for development of high-density genetic map and QTL mapping of Ascochyta blight resistance in chickpea (*Cicer arietinum* L.). Theor. Appl. Genet..

[27] Sab S (2020). Genome-wide SNP discovery and mapping QTLs for seed iron and zinc concentrations in chickpea (*Cicer arietinum* L.). Front. Nutr..

[28] Barmukh R (2020). Development of a dense genetic map and QTL analysis for pod borer *Helicoverpa armigera* (Hübner) resistance component traits in chickpea (*Cicer arietinum* L.). Plant Genome.

[29] Barmukh R (2021). Construction of a high-density genetic map and QTL analysis for yield, yield components and agronomic traits in chickpea (*Cicer arietinum* L.). Plos One.

[30] Jha UC (2021). Major QTLs and potential candidate genes for heat stress tolerance identified in chickpea (*Cicer arietinum* L.). Front. Plant Sci..

[31] Jansen RC, Nap JP (2001). Genetical genomics: The added value from segregation. Trends Genet..

[32] Mackay TF (2014). Epistasis and quantitative traits: Using model organisms to study gene–gene interactions. Nat. Rev. Genet..

[33] Aranjuelo I (2013). Harvest index, a parameter conditioning responsiveness of wheat plants to elevated CO2. J. Exp. Botany.

[34] El-Soda M (2014). Genotype × environment interaction QTL mapping in plants: Lessons from Arabidopsis. Trends Plant Sci..

[35] Li Y (2018). Investigating drought tolerance in chickpea using genome-wide association mapping and genomic selection based on whole-genome resequencing data. Front. Plant Sci..

[36] Monneveux P, Sanchez C, Beck D, Edmeades G (2006). Drought tolerance improvement in tropical maize source populations. Crop Sci..

[37] Cai C, Zhu G, Zhang T, Guo W (2017). High-density 80K SNP array is a powerful tool for genotyping *G. hirsutum* accessions and genome analysis. BMC Genomics.

[38] Senthilvel S, Ghosh A, Shaik M, Shaw RK, Begali PG (2019). Development and validation of an SNP genotyping array and construction of a high-density linkage map in castor. Sci. Rep..

[39] Jiang SC, Mei C, Liang S (2015). Crucial roles of the pentatricopeptide repeat protein SOAR1 in *Arabidopsis* response to drought, salt and cold stresses. Plant Mol. Biol..

[40] Gaur R (2012). High-throughput SNP discovery and genotyping for constructing a saturated linkage map of chickpea (*Cicer arietinum* L.). DNA Res..

[41] Hiremath PJ (2012). Large-scale development of cost-effective SNP marker assays for diversity assessment and genetic mapping in chickpea and comparative mapping in legumes. Plant Biotechnol. J..

[42] Deokar AA (2014). Genome wide SNP identification in chickpea for use in development of a high-density genetic map and improvement of chickpea reference genome assembly. BMC Genomics.

[43] Gowda CLL, Upadhyaya HD, Dronavalli N, Singh S (2011). Identification of large-seeded high-yielding stable Kabuli chickpea germplasm lines for use in crop improvement. Crop Sci..

[44] Saxena MS (2014). An integrated genomic approach for rapid delineation of candidate genes regulating agro-morphological traits in chickpea. DNA Res..

[45] Minocha R, Majumdar R, Minocha SC (2014). Polyamines and abiotic stress in plants: A complex relationship. Front. Plant Sci..

[46] Garg R (2016). Transcriptome analyses reveal genotype- and developmental stage-specific molecular responses to drought and salinity stresses in chickpea. Sci. Rep..

[47] Tenhaken R (2015). Cell wall remodeling under abiotic stress. Front. Plant Sci..

[48] Jain M, Khurana JP (2009). Transcript profiling reveals diverse roles of auxin-responsive genes during reproductive development and abiotic stress in rice. FEBS J..

[49] Peleg Z, Blumwald E (2011). Hormone balance and abiotic stress tolerance in crop plants. Curr. Opin. Plant Biol..

[50] Kumar PP (2013). Regulation of biotic and abiotic stress responses by plant hormones. Plant Cell Rep..

[51] Kumar M (2019). Transcriptome Sequencing of Chickpea (*Cicer arietinum* L.) genotypes for identification of drought-responsive genes under drought stress condition. Plant Mol. Biol. Rep..

[52] Wu J (2020). Resequencing of 683 common bean genotypes identifies yield component trait associations across a north-south cline. Nat. Genet..

[53] Maeda H, Dudareva N (2012). The shikimate pathway and aromatic amino acid biosynthesis in plants. Ann. Rev. Plant Biol..

[54] Agati G, Azzarello E, Pollastri S, Tattini M (2012). Flavonoids as antioxidants in plants: location and functional significance. Plant Sci..

[55] Rao MJ (2020). CsCYT75B1, a Citrus CYTOCHROME P450 gene, is involved in accumulation of antioxidant flavonoids and induces drought tolerance in transgenic Arabidopsis. Antioxidants.

[56] Tohge T, Watanabe M, Hoefgen R, Fernie AR (2013). The evolution of phenylpropanoid metabolism in the green lineage. Crit. Rev. Biochem. Mol. Biol..

[57] Lattanzio V, Lattanzio VMT, Cardinali A (2006). Role of phenolics in the resistance mechanisms of plants against fungal pathogens and insects. Phytochem. Adv. Res..

[58] Saghai-Maroof MA, Soliman KM, Jorgensen RA, Allard RW (1984). Ribosomal DNAsepacer-length polymorphism in barley: Mendelian inheritance, chromosomal location, and population dynamics. Proc. Nat. Acad. Sci..

[59] Bradbury PJ (2007). TASSEL: Software for association mapping of complex traits in diverse samples. Bioinformatics.

[60] Browning BL, Browning SR (2008). A unified approach to genotype imputation and haplotype-phase inference for large data sets of trios and unrelated individuals. Am. J. Human Genet..

[61] Krzywinski M (2009). Circos: An information aesthetic for comparative genomics. Genome Res..

[62] Cingolani P (2012). A program for annotating and predicting the effects of single nucleotide polymorphisms, SnpEff: SNPs in the genome of Drosophila melanogaster strain w1118; iso-2; iso-3. Fly.

[63] Shannon P (2003). Cytoscape: A software environment for integrated models of biomolecular interaction networks. Genome Res..

[64] Van Ooijen JW (2011). Multipoint maximum likelihood mapping in a full-sib family of an outbreeding species. Genet. Res..

[65] Isidore E (2003). Toward a marker-dense meiotic map of the potato genome: Lessons from linkage group I. Genetics.

[66] Kosambi DD (1944). The estimation of map distances from recombination values. Ann. Eugen..

[67] Voorrips RE (2002). MapChart: Software for the graphical presentation of linkage maps and QTLs. J. Hered..

[68] Zhang YW, Wen YJ, Dunwell JM, Zhang YM (2020). QTL.gCIMapping.GUI v2.0: An R software for detecting small-effect and linked QTLs for quantitative traits in bi-parental segregation populations. Comput. Struct. Biotechnol. J..

[69] Solovyev V, Kosarev P, Seledsov I, Vorobyev D (2006). Automatic annotation of eukaryotic genes, pseudogenes and promoters. Genome Biology.

[70] Mashaki KM (2018). RNA-Seq analysis revealed genes associated with drought stress response in kabuli chickpea (*Cicer arietinum* L.). PLoS One.

[71] Saeed AI (2003). TM4: A free, open-source system for microarray data management and analysis. BioTechniques.

[72] Saeed AI (2006). TM4 microarray software suite. Methods Enzymol..

[73] Conesa A (2005). Blast2GO: A universal tool for annotation, visualization and analysis in functional genomics research. Bioinformatics.

[74] Barrett JC, Fry B, Maller J, Daly MJ (2005). Haploview: Analysis and visualization of LD and haplotype maps. Bioinformatics.

