# Supplementary material for: Genetic mapping of quantitative trait loci associated with drought tolerance in chickpea (Cicer arietinum L.)
Source: Sci Rep. 2023 Oct 17;13:17623. doi: 10.1038/s41598-023-44990-y (PMC10582051; doi:10.1038/s41598-023-44990-y)
Supplement: Supplementary file 2 — Supplementary Tables. [file 41598_2023_44990_MOESM2_ESM.docx]

**Genetic mapping of quantitative trait loci associated with drought tolerance in chickpea (*Cicer arietinum* L.)**

**Yashwant K. Yadava^1^, Pooja Chaudhary^1^, Sheel Yadav^1^, Aqeel Hasan Rizvi^2^, Tapan Kumar^2^, Rachna Shrivastava^2^, KR Soren^3^, Bharadwaj C^2^, R Srinivasan^1^, NK Singh^1^, PK Jain^1^**

**^1^** ICAR-National Institute for Plant Biotechnology, IARI Campus, New Delhi 110012

^2^ ICAR-Indian Agricultural Research Institute, Division of Genetics, New Delhi 110012

^3^ICAR-Indian Institute of Pulses Research, Kanpur 208024

***Corresponding author e - mail** : [jainpmb@gmail.com](mailto:jainpmb@gmail.com)

**ORCID ID (P.K. Jain) : https://orcid.org/0000-0001-5199-4429**


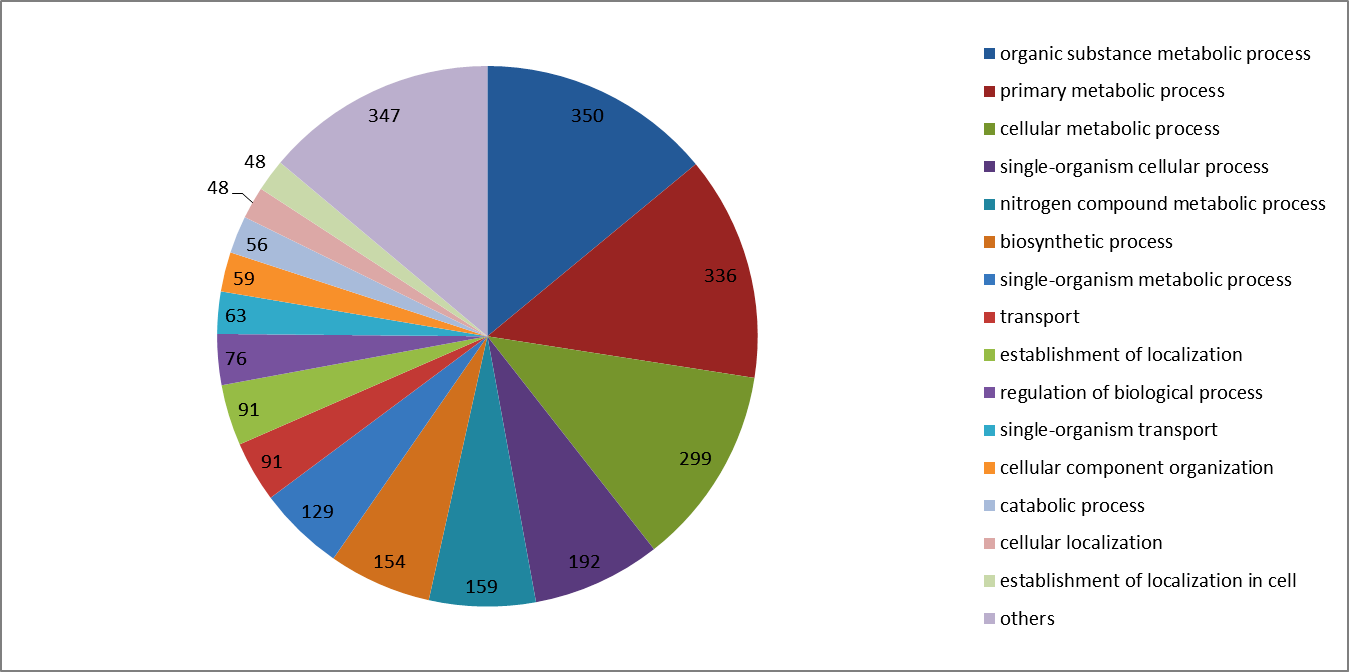
 **a.**


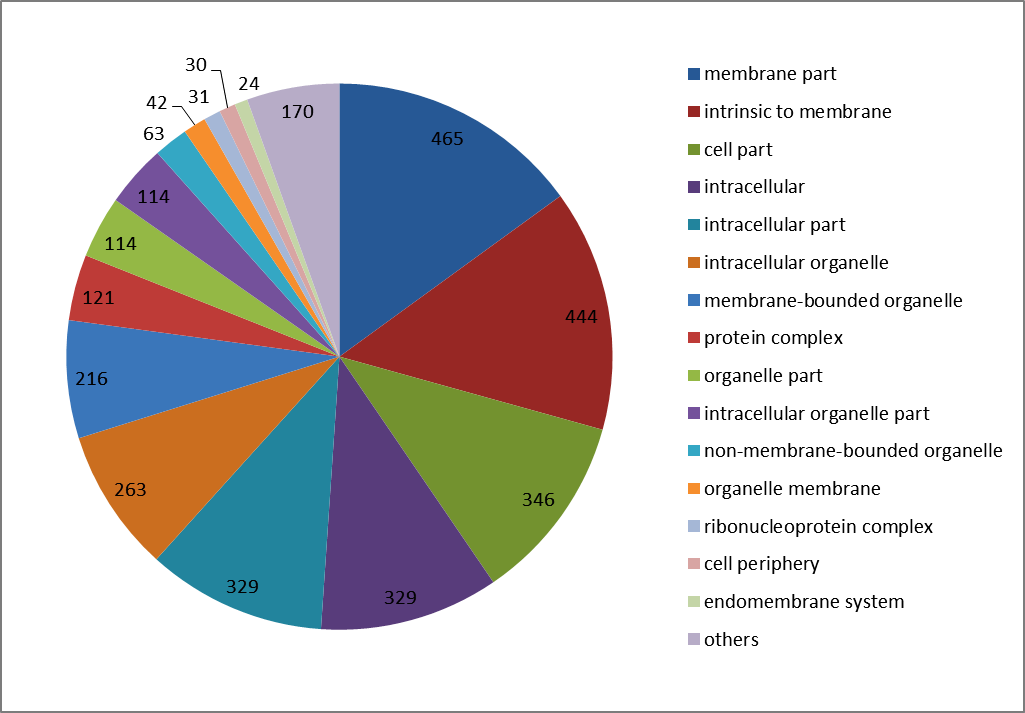
 **b.**


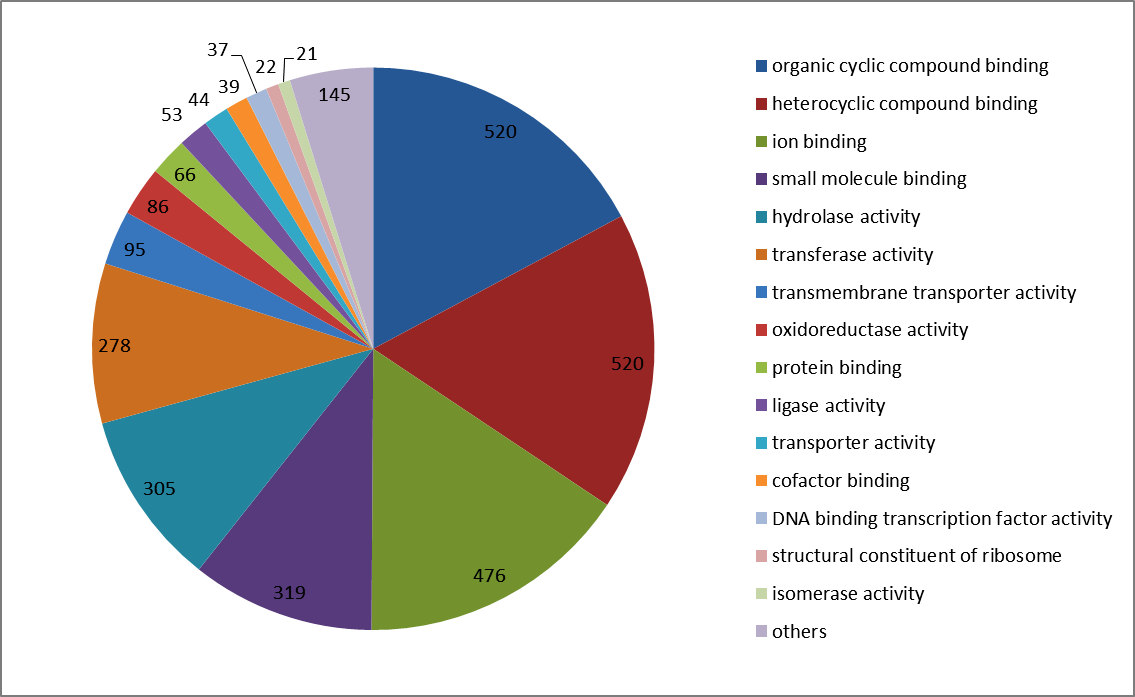
 **c.**

**Supplementary Figure S1.** Functional annotation of genes carrying genic SNPs. a. Biological, b. Cellular, c. Molecular


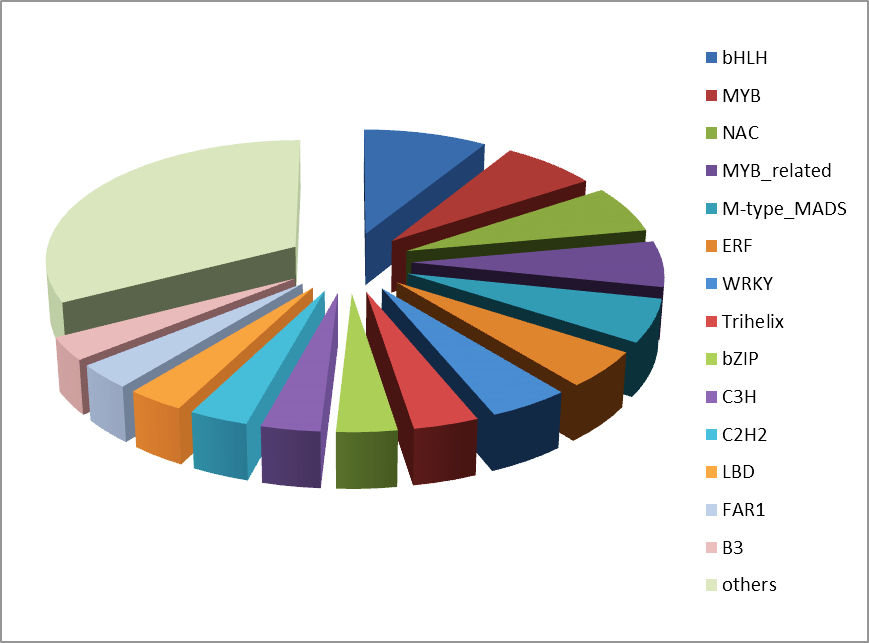
**Supplementary Figure S2.** Distribution of genes carrying genic SNPs into transcription factor families.

**Supplementary Table S1:** SNPs with their flanking sequences

(Provided as a separate MS Excel file, sheet 1)

**Supplementary Table S2:** Number and frequency of SNPs detected on individual chickpea chromosomes

(Provided as a separate sheet in the above mentioned Excel file)

**Supplementary Table S3:** Structural and functional annotation of SNPs

(Provided as a separate sheet in the above mentioned Excel file)

**Supplementary Table S4:** Genetic positions of 3237 markers distributed on eight linkage groups of chickpea intra-specific linkage map

(Provided as a separate sheet in the above mentioned Excel file)

**Supplementary Table S5:** List of candidate genes and their expression values (RPKM) in diferent tissue with drought specific expression

(Provided as a separate sheet in the above mentioned Excel file)
